# A New Insight into the Mechanisms Underlying the Discoloration, Sorption, and Photodegradation of Methylene Blue Solutions with and without BNO_x_ Nanocatalysts

**DOI:** 10.3390/ma15228169

**Published:** 2022-11-17

**Authors:** Andrei T. Matveev, Liubov A. Varlamova, Anton S. Konopatsky, Denis V. Leybo, Ilia N. Volkov, Pavel B. Sorokin, Xiaosheng Fang, Dmitry V. Shtansky

**Affiliations:** 1 Research Laboratory Inorganic Nanomaterials, National University of Science and Technology (MISIS), Leninskiy Prospect 4, 119049 Moscow, Russia; 2 Department of Materials Science, Fudan University, Shanghai 200433, China

**Keywords:** methylene blue, discoloration, sorption, photodegradation, BNO_x_ nanocatalyst, DFT calculations

## Abstract

Methylene blue (MB) is widely used as a test material in photodynamic therapy and photocatalysis. These applications require an accurate determination of the MB concentration as well as the factors affecting the temporal evolution of the MB concentration. Optical absorbance is the most common method used to estimate MB concentration. This paper presents a detailed study of the dependence of the optical absorbance of aqueous methylene blue (MB) solutions in a concentration range of 0.5 to 10 mg·L^−1^. The nonlinear behavior of optical absorbance as a function of MB concentration is described for the first time. A sharp change in optical absorption is observed in the range of MB concentrations from 3.33 to 4.00 mg·L^−1^. Based on the analysis of the absorption spectra, it is concluded that this is due to the formation of MB dimers and trimers in the specific concentration range. For the first time, a strong, thermally induced discoloration effect of the MB solution under the influence of visible and sunlight was revealed: the simultaneous illumination and heating of MB solutions from 20 to 80 °C leads to a twofold decrease in the MB concentration in the solution. Exposure to sunlight for 120 min at a temperature of 80 °C led to the discoloration of the MB solution by more than 80%. The thermally induced discoloration of MB solutions should be considered in photocatalytic experiments when tested solutions are not thermally stabilized and heated due to irradiation. We discuss whether MB is a suitable test material for photocatalytic experiments and consider this using the example of a new photocatalytic material—boron oxynitride (BNO_x_) nanoparticles—with 4.2 and 6.5 at.% of oxygen. It is shown that discoloration is a complex process and includes the following mechanisms: thermally induced MB photodegradation, MB absorption on BNO_x_ NPs, self-sensitizing MB photooxidation, and photocatalytic MB degradation. Careful consideration of all these processes makes it possible to determine the photocatalytic contribution to the discoloration process when using MB as a test material. The photocatalytic activity of BNO_x_ NPs containing 4.2 and 6.5 at.% of oxygen, estimated at ~440 μmol·g^−1^·h^−1^. The obtained results are discussed based on the results of DFT calculations considering the effect of MB sorption on its self-sensitizing photooxidation activity. A DFT analysis of the MB sorption capacity with BNO_x_ NPs shows that surface oxygen defects prevent the sorption of MB molecules due to their planar orientation over the BNO_x_ surface. To enhance the sorption capacity, surface oxygen defects should be eliminated.

## 1. Introduction

Methylene blue (MB) is a widely used phenothiazinium dye that finds applications as a photosensitizer, as well as a redox and optical redox indicator in analytical chemistry and in trace analyses of anionic surfactants [1]. MB is also used for anticancer treatments in photodynamic therapy [2,3,4,5,6]. The widespread industrial use of dyes leads to their inevitable release into the environment. According to available estimates, about 10–15% of more than 0.7 million tons of dyes produced annually worldwide are released into the environment [7]. At the same time, MB is known to be a toxic and carcinogenic pollutant, which requires precise control over its concentration, as well as efficient removal or degradation to less toxic substances. Adsorption and photodegradation are effective ways to purify water from toxic pollutants. Various materials, such as activated carbon and coal [8,9], as well as natural and renewable biomaterials [10,11,12], are used as adsorbents for MB removal. Various adsorbents for organic and inorganic substances have been tested [13,14,15]. Recently, it was shown that hexagonal boron nitride (*h*-BN) is also a good adsorbent for organics [16,17,18].

The discoloration of dye solutions is one of the main methods of studying adsorption and photodegradation, and MB dye is a widely used test material for these reactions. For MB photodegradation, a wide variety of nanocatalytic assemblies, mainly consisting of binary and ternary metal oxides, have been studied [19,20]. A number of publications note that MB is not a suitable test material for photocatalytic experiments, since it becomes colorless when irradiated with visible light [21,22]. On the one hand, the self-photodiscoloration of MB contributes to its degradation and reduces the severity of the problem of its accumulation in the environment. On the other hand, the self-photodegradation of MB should be taken into account to avoid errors in the assessment of the photocatalytic activity of the studied photocatalyst. This requires a deep understanding of the photo decolorization of MB solutions.

It has recently been shown that boron oxynitride (BNO_x_) nanoparticles (NPs) are good substrates for photocatalysis under UV irradiation [20]. BNO_x_ is a relatively cheap, chemically inert, and environmentally friendly material. Here, we studied its photocatalytic activity using MB as a test material. BNO_x_ NPs containing 4.2 and 6.5 at.% of oxygen were studied as photocatalysts for MB degradation under UV illumination. The main objectives of the study were (i) a detailed study of the optical absorbance of aqueous MB solutions at various concentrations; (ii) a study of the self-discoloration effect of MB solutions (including heating) under visible and artificial sunlight illumination; (iii) an analysis of the applicability of MB as a test material for photocatalytic experiments; (iv) an investigation of the photocatalytic activity of BNO_x_ NPs in the photodegradation of MB solutions under UV illumination; (v) a study of the effect of oxygen on the photocatalytic and sorption capacity of BNO_x_ NPs; (vi) an analysis of the stability of BNO_x_ NPs and the possibility of their reuse in photocatalytic experiments; (vii) and to answer the question of whether MB is a suitable test material for photocatalytic experiments.

It has been shown that the discoloration of MB solutions is a complex process involving the following mechanisms: thermally induced MB photodegradation, MB absorption on BNO_x_ NPs, self-sensitizing MB photooxidation, and photocatalytic MB degradation. Taking into account all of these mechanisms, the photocatalytic activity of BNO_x_ NPs containing 4.2 and 6.5 at.% of oxygen is estimated to be as high as ~440 μmol·g^−1^·h^−1^. The obtained results are discussed based on DFT calculations, taking into account the effect of MB sorption on its self-sensitizing photooxidation activity.

## 2. Materials and Methods

### 2.1. Materials

MB in the form of a hydrochloride salt (with three water molecules) was acquired from Rushim (Moscow, Russia). MB has a molecular weight of 319.85 g·mol^−1^. MB is a cationic thiazine dye with the molecular formula C_16_H_18_N_3_ClS. It is highly water-soluble and forms a stable solution with water at room temperature. MB has an amino autochrome unit and has a maximum of optical absorption absorbance at of 663 nm [1]. BNO_x_ NPs with various oxygen concentrations were synthesized with the low-temperature ammonolysis of boric acid, as described elsewhere [23]. Boric acid was treated with gaseous ammonia at room temperature to produce an ammonium borate hydrate (ABH) phase. Heating the ABH phase in ammonia led to successive dehydration and, starting from a temperature of 550 °C, the formation of *h*-BNO_x_ NPs. The size of the resulting BNO_x_ NPs, as well as the oxygen content, depended on the maximum heating temperature. BNO_x_ NPs synthesized at 650 °C were designated sample BNO_1_. The *h*-BNO_x_ nanopowder, further annealed in ammonia at 1100 °C for 1 h, was designated sample BNO_2_.

### 2.2. Materials Characterization

The sample phase composition was determined with a SmartLab diffractometer (Rigaku, Tokyo, Japan) using Cu-Kα radiation and a graphite monochromator. X-ray diffraction (XRD) patterns were recorded in symmetrical mode and analyzed using the PDXL software, (Version 2.8.4.0) (Rigaku, Tokyo, Japan). Fourier-transform infrared (FTIR) spectra were recorded based on powder samples using a Vertex 70v vacuum spectrometer (Bruker, Billerica, MA, USA) in the range of 400–4000 cm^−1^ with a partial internal reflection device. The chemical composition was analyzed with an X-ray photoelectron spectroscopy (XPS, 18725 Lake Drive East, Chanhassen, MN, USA) using a Versa Probe III (PHI) instrument equipped with a monochromatic Al Kα X-ray source (*hν* = 1486.6 eV). Atomic concentrations were determined from survey spectra using the relative sensitivity factors of the elements. The integral intensities of the XPS B1s, N1s, O1s, and C1s peaks were used for analysis. The specific surface area was determined with the Brunauer–Emmett–Teller (BET) nitrogen adsorption method using a NOVA 1200e instrument (Quantachrome Instruments, Boynton Beach, FL, USA).

### 2.3. Spectrophotometric Measurements

The ultraviolet–visible (UV-vis) absorption spectra were recorded on an UVmini-1240 spectrophotometer (Shimadzu, Tokyo, Japan) using a 1 cm quartz cuvette. Fluorescence spectra were recorded on a Cary Eclipse fluorescence spectrophotometer (Agilent Technologies, Santa Clara, CA, USA).

Diffuse reflectance spectra in the ultraviolet and visible regions (DRS UV-vis) were recorded on a V-750 spectrophotometer (Jasco, JASCO Corporation, Tokyo, Japan) in the wavelength of 200–800 nm with a resolution of 1 nm. Barium sulfate was used as a standard.

### 2.4. Discoloration and Photocatalytic Measurements

The discoloration and photodegradation of MB aqueous solutions were studied under UV, solar, and visible light. A 50 W low-pressure mercury lamp with a main emission line at a wavelength of 254 nm was used as a UV source. An Osram Ultra-Vitalux lamp (Munich, Germany) was employed as a source of simulated sunlight. For visible light illumination, a filter was used that cuts off the UV part of the spectrum with wavelengths shorter than 420 nm. 

For photocatalytic measurements under visible and sunlight illumination, 10 mg of BNO_1_ NPs were ultrasonically dispersed in 25 mL of distilled water, and then 25 mL of an MB aqueous solution was added. The MB concentration in the resulting solution was 10 mg·L^−1^. The experiments were carried out in Duran glasses. The same protocol was used for photocatalytic measurements under UV irradiation, with the only difference being that quartz glasses and 5 mg of BNO_1_ or BNO_2_ nanopowders were used for each solution. When the solution was illuminated, its temperature increased. A water-cooled cell was used to stabilize the solution temperature at 20 or 80 °C. The solution temperature was controlled by a thermocouple. The loss of water due to illumination-induced evaporation was compensated after each illumination stage before taking an aliquot for analysis. The catalyst mass-specific activity was calculated as the number of moles of degraded MB divided by the weight of the catalyst and the degradation time. To evaluate the stability and reusability of the BNO_x_ nanocatalysts under UV illumination, four successive photocatalytic cycles were performed accordingly to the above protocol. After each cycle, the solution was centrifuged at 9000 rpm for 15 min, the supernatant was taken with a syringe, and fresh MB solution was added.

The following chemicals were used as scavengers: isopropyl alcohol (IPA) as an **·**OH scavenger, dimethyl sulfoxide as an electron (e**^−^**) scavenger, disodium ethylenediaminetetraacetate (Na-EDTA) to remove positive charge carriers (h^+^), and benzoquinone to neutralize superoxide anion **^-^**O**˙**_2_. The scavengers tests were performed for 20 min of UV illumination of MB solutions (50 mL with an MB concentration of 10 mg·L^−1^) containing 5 mg of BNO_1_ NPs.

### 2.5. Computational Methods

Density functional theory (DFT) calculations were performed using the VASP package with the PBE functional and a plane wave cutoff of 400 eV [24,25,26]. Spin-polarized calculations were used. During relaxation, the atomic positions and lattice parameters were optimized.

## 3. Results and Discussion

### 3.1. Spectrophotometry of MB Solutions

**Figure 1:**
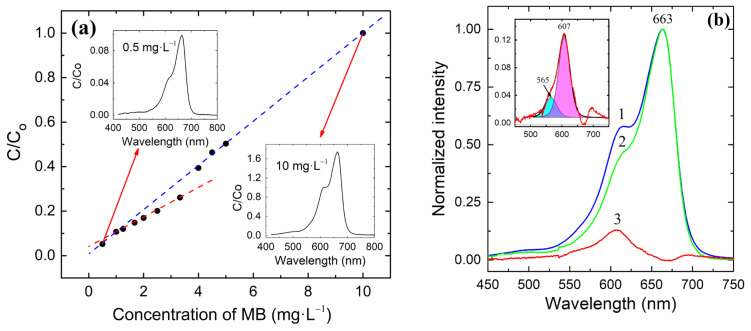
Concentration dependence of the normalized absorbance of MB aqueous solutions in a concentration range of 0.5 to 10 mg·L^−1^ (from 1.56 × 10^−6^ to 3.13 × 10^−5^ mol·L^−1^) at 25 °C (**a**). The insets in (**a**) show absorbance spectra at the lowest and highest measured concentrations. Normalized absorbance of MB solution (**b**): 10 mg·L^−1^ (curve 1) and 0.5 mg·L^−1^ (curve 2). Curve 3 represents the difference spectrum obtained by subtracting curve 2 from curve 1. Inset in (**b**) shows the deconvolution of difference spectrum 3.

The concentration of dyes in a solution is most often determined by light absorption using the spectroscopic method. The MB molecule is planar and exists as a cation in aqueous solutions. At a certain concentration, MB molecules tend to form sandwich-like dimers, trimers, or higher oligomers [27], also called H-type aggregates. Quite recently, it was suggested that the MB molecule exists in two mesomere forms, which differ in their electric charge distribution [28]. The MB monomer, dimer, trimer, n-oligomers, and mesomeres have different molar attenuation coefficients; therefore, it can be expected that a change in their solution concentration can lead to a nonlinear dependence of solution optical absorption on MB concentration. Figure 1a shows the concentration dependence of the normalized optical absorption of MB aqueous solutions in an MB concentration range of 0.5 to 10 mg·L^−1^ (from 1.56 × 10^−6^ to 3.13 × 10^−5^ mol·L^−1^). Here and below, C_0_ is the absorbance of the initial MB solution, and C is the absorbance at a given time of illumination.

In the entire range of studied concentrations, the dependence is not linear. At concentrations above 1.0 mg·L^−1^, the absorbance deviates from the initial trend and follows a line with a lower slope up to 3.33 mg·L^−1^. Between 3.33 and 4.00 mg·L^−1^, the C_t_/C_o_ value sharply increases (dotted area in Figure 1a), and then, the optical absorbance follows a line with approximately the same slope as it does at low concentrations ranging from 0.5 to 1.0 mg·L^−1^.

To identify the cause of optical absorption deviating from linearity in the range of 3.33–4.00 mg·L^−1^, a normalized absorption spectrum for the 0.5 mg·L^−1^ solution (curve 1 in Figure 1b) was subtracted from the normalized absorption spectrum of the 10 mg·L^−1^ solution (curve 2). The spectrum obtained after subtraction (curve 3) was fitted using two components at 607 nm and 565 nm. These peaks almost coincide with those reported for the dimer [29] and trimer [29,30], respectively. Thus, we can conclude that the observed deviation of the concentration curve from the linear Beer–Lambert law is due to the formation of dimeric and trimeric molecular associates. The fractions of the monomer, dimer, and trimer in the 10 mg·L^−1^ MB solution, estimated from the peak areas, are 91.1%, 7.1%, and 1.8%, respectively. To the best of our knowledge, this is the first mention of the nonlinearity of the optical absorption of MB solutions in a low concentration range. An additional analysis of the data presented in [29] also shows a change in the relative content of the monomers, dimers, and trimers, but the authors did not pay attention to this fact. The trimer fraction increases with the increasing MB concentration in two steps: first, a small step above approximately 0.5 × 10^−5^ mol·L^−1^ (determined from Figure 1 in [29]), and then, a second strong step in a range of 1.2 × 10^−5^ to 4.5 × 10^−5^ mol·L^−1^, which correlates well with the step observed in Figure 1. The fraction of the monomer accordingly decreases stepwise with the increasing MB concentration.

It should be noted that the available data on MB agglomeration and polymerization are rather contradictory. The presence of at least three absorbing species (monomers, dimers, and trimers) has been observed in a concentration range of 6.0 × 10^−7^ mol·L^−1^ to 6.0 × 10^−2^ mol·L^−1^, and it was suggested that trimerization occurs simultaneously with dimerization due to the reaction of dimers with monomers [30]. Heger et al. observed a very small fraction of dimers, but also a steadily increasing fraction of trimers above 1.0 × 10^−5^ mol·L^−1^ [29]. It has recently been shown that the fraction of dimers increases from 1.0 × 10^−6^ mol·L^−1^, reaches a maximum at approximately 1.0 × 10^−4^ mol·L^−1^, and then decreases, while the fraction of tetramers and oligomers constantly increases above 1.0 × 10^−6^ mol·L^−1^, but trimers were not observed [27]. Therefore, it would be very speculative to propose a detailed model explaining the nonlinear behavior of absorbance as a function of MB concentration. For this, additional studies of MB agglomeration and polymerization are required. In addition, accurate measurements of the molar attenuation coefficients of each n-dimensional MB type are required.

It should also be noted that we did not observe an absorption peak with a maximum at 600 nm, observed elsewhere and attributed to the tetramer [27].

It is important to note that the error in estimating the MB concentration from the optical absorption tests reaches 1 mg·L^−1^ without taking into account the change in the slope of the concentration curve. Given the toxicity of MB, such an error may be important in some applications, such as biomedicine.

**Figure 2:**
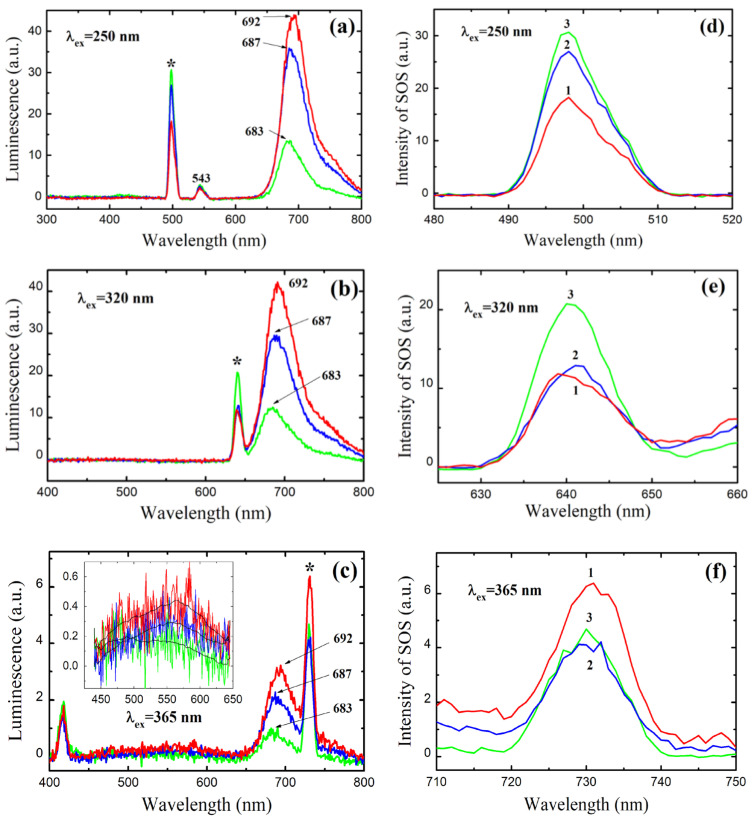
Luminescence spectra (**a**–**c**) of MB aqueous solutions with MB concentrations of 1 mg·L^−1^ (green line), 3.33 mg·L^−1^ (blue line), and 5 mg·L^−1^ (red line) under excitation wavelengths of 250 (**a**), 320 (**b**), and 365 (**c**) nm. Asterisks mark second-order scattering. Panels (**d**–**f**) show enlarged peaks of second-order scattering; numbers 1, 2, and 3 correspond to MB concentrations of 5, 3.33, and 1 mg·L^−1^.

To further explain the observed feature of the absorption/concentration curve, we obtained and analyzed the luminescence spectra of the three solutions (with MB concentrations of 1, 3.33, and 5.0 mg·L^−1^ near the curve inflection) at three excitation wavelengths: 250, 320, and 365 nm (Figure 2). At an excitation wavelength of 250 nm and an MB concentration of 1 mg·L^−1^, the luminescence peak is observed at 683 nm. This is a characteristic MB emission [31]. With an increase in the MB concentration, the peak intensity increases, and its position shifts by 4–9 nm: 683 nm at 1 mg·L^−1^, 687 nm at 3.33 mg·L^−1^, and 692 nm at 5 mg·L^−1^. This behavior is observed for all studied excitation wavelengths. We consider these results as additional evidence of oligomer formation since it is known that the fluorescence lines of dimers shift toward longer wavelengths relative to the fluorescence line of the monomeric form [32]. In addition to a peak at approximately 690 nm, MB exhibits a strong and broad fluorescence zone in a range of 450–650 nm when excited at a wavelength of 365 nm [33]. Fluorescence at 690 nm was associated with electron transitions from dimethyl amino groups to the central aromatic ring, i.e., along the longer molecule axis, while fluorescence at 550 nm was assigned to a transition along the shorter molecule axis, when electrons from sulfur move to nitrogen [33]. In the range of 450–650 nm, we observed only a very weak luminescence zone (inset in Figure 2c), which indicates the almost complete absence of an electron transition between sulfur and nitrogen (in contrast to [28,33]). This can be explained by the lower MB concentration (by one order of magnitude) used in our studies. It is important to note that the emission of *leuco*-MB at 460 nm upon excitation at a wavelength of 320 nm was not observed [31]. This eliminates the possibility that the abrupt increase in absorbance is due to the oxidation of colorless *leuco*-MB to color MB. The narrow peaks observed at 543 nm (excitation at 250 nm) and at ~420 nm (excitation at 365 nm) are insensitive to MB concentration and have a full width at a half maximum (FWHM) value of approximately 15 nm, which is typical for the Raman scattering of light in water (Figure 2a,c) [34].

Additional narrow and strong peaks are observed at 500 nm, 640 nm, and 730 nm when excited at wavelengths of 250, 320, and 365 nm, respectively. These peaks are marked with asterisks in Figure 2a–c and are also shown at enlarged scales in Figure 2d–f. The intensity of these peaks depends on the MB concentration and the excitation wavelength. We assume that these maxima are due to the second-order scattering (SOS) of light, as they are observed at wavelengths twice that of the excitation wavelength. Indeed, at an excitation wavelength of 250 nm (Figure 2d), all peaks are observed at 498 nm; i.e., they are shifted by 2 nm toward a shorter wavelength. In the past, SOS was commonly observed in spectroscopic measurements and was considered a kind of interference phenomenon until it was shown that the SOS intensity of the aqueous solution of the ion-association complex of the Se (IV)–I^−^-rhodamine B system is sensitive to trace amounts of Se [35]. Since then, SOS peaks have been successfully used to study the structure and concentration of various colloids, including macromolecules, nanoparticles, quantum dots, and, especially, organic polymers such as proteins [36,37,38,39,40].

In contrast to luminescence (Figure 2a), at 250 nm excitation, the intensity of the SOS peak at 500 nm demonstrates an inverse dependence on the MB concentration (Figure 2d). A similar effect was observed at a strong dilution of humate solutions and was explained by an increase in the number of scattering centers upon dilution [40]. In this regard, the observed reverse order of SOS intensities suggests an association of MB molecules at a low MB concentration. With an increase in the MB concentration, as discussed above, oligomers are formed, and associates are destroyed due to the steric effect. More reliable conclusions require additional SOS spectroscopic studies of MB solutions at various concentrations.

### 3.2. Discoloration of MB Solutions under Visible Light and Sunlight

**Figure 3:**
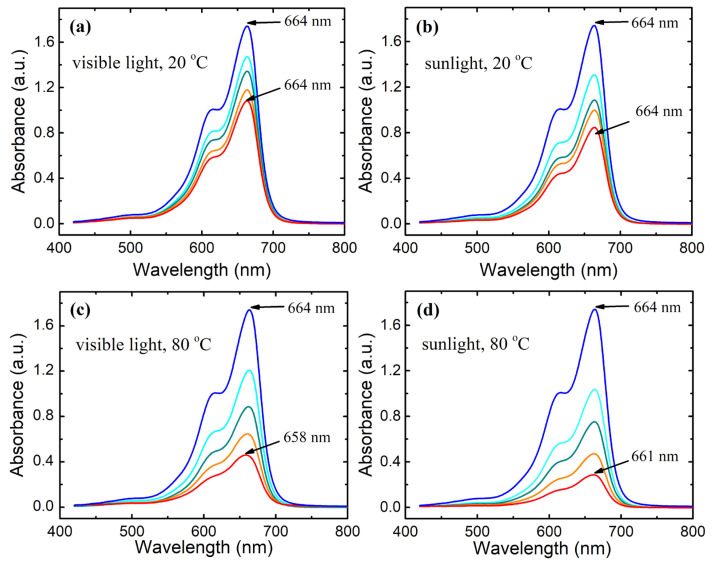
Absorption spectra of MB aqueous solution with a concentration of 10 mg·L^−1^ under visible light and sunlight illumination at temperatures of 20 and 80 °C for 120 min. Lighting time from upper blue curve to lower red curve: 0, 30, 60, 90, and 120 min. (**a**) illumination by visible light at 20 °C; (**b**) illumination by sunlight at 20 °C; (**c**) illumination by visible light at 80 °C; (**d**) illumination by sunlight at 80 °C.

An MB aqueous solution with a concentration of 10 mg·L^−1^ was exposed to visible light and sunlight at temperatures of 20 and 80 °C for 120 min. The solution absorption spectra are shown in Figure 3. When illuminated, the intensities of the main absorbance peak located at 664 nm and the shoulder at approximately 600 nm decrease. The position of the main peak does not change at 20 °C but slightly shifts toward a shorter wavelength at 80 °C, demonstrating a weak hypsochromic effect, more pronounced under visible light. These changes in the absorbance spectra suggest the degradation of chromophore moieties in the MB molecule.

**Figure 4:**
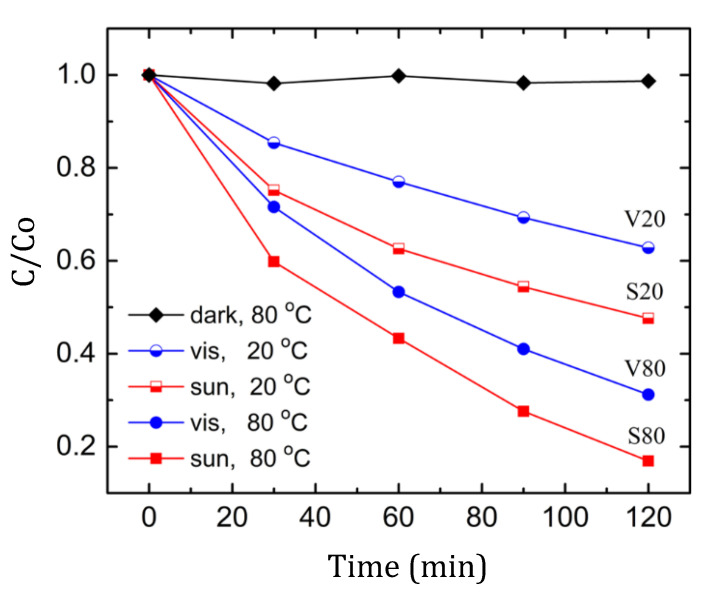
Temporal changes in the normalized absorbance of MB solution (10 mg·L^−1^) in the dark at 80 °C (black symbols) and under illumination with visible (V) light and sunlight (S) at 20 and 80 °C.

The time dependences of the normalized optical absorbance of the MB solutions are depicted in Figure 4. For comparison, the normalized absorbance values of the MB solution heated in the dark to 80 °C are shown with black symbols. Heating the MB solution in the dark did not affect its color. Illuminating the MB solution at 20 °C for 120 min led to a decrease in absorption by 37% (V20) and 53% (S20). When the solution temperature was raised to 80°C, the absorption decreased by 69% (V80) and 83% (S80). Thus, the MB solution rapidly decolorized under visible light and even more rapidly under sunlight (containing some UV component), with decolorization greatly accelerated with the increasing temperature. The decolorization rate (observed from the curve slopes) also increased with heating, and the absorption curves do not tend toward any asymptotic limit. To the best of our knowledge, this is the first observation of strong thermal photodegradation in an MB solution. MB thermal degradation with an efficiency of more than 80% has been reported, but only in the presence of a catalyst [41]. Thus, when studying the photocatalytic degradation of MB solutions, it is necessary to take into account the self-decomposition of MB under visible light and sunlight in order to avoid an incorrect assessment of the material photocatalytic activity if the temperature of the solutions is unstable and increases with prolonged illumination.

It is generally accepted that the photodecomposition of organic compounds occurs as a result of their interaction with active species formed during the light-activated process. Most often, such species are hydroxyl (**·**OH), superoxide (**·**O_2_^−^), and peroxide (HO_2_**·**) radicals, as well as holes h^+^ [42]. The positions of the LUMO and HOMO of MB were estimated as −0.88 and 1.55 eV, respectively [43]. The MB bandgap is 2.43 eV, and visible light photons can transfer energy to electrons and facilitate their transition from HOMO to LUMO orbitals. Thus, MB can be used as a photosensitizer in various applications, including phototherapy [2,3,4,5,6,44] and water photo-splitting [45]. In the visible-light-driven self-decomposition process, MB apparently acts as a self-photosensitizer. The formation of MB oligomers and mesomeres, as well as the temperature change in the dielectric constant of water [28], provokes a change in the electron charge distribution in the MB monomer and affects this photodecomposition process.

**Figure 5:**
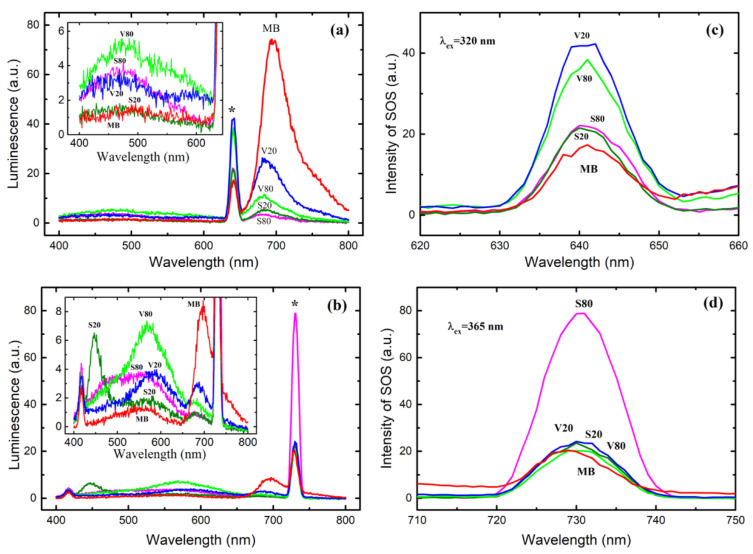
Luminescence spectra upon excitation at wavelengths of 320 nm (**a**) and 365 nm (**b**) of MB aqueous solutions (10 mg·L^−1^) after illumination with visible (V) light and sunlight (S) at 20 °C and 80 °C for 120 min. Asterisks mark second-order scattering. The insets show magnified parts of the spectra. The enlarged second-order scattering peaks are (**c**) and (**d**).

To understand the MB photodegradation process, the solution fluorescence spectra were obtained at excitation wavelengths of 320 and 365 nm after solution illumination with visible light and sunlight for 120 min (Figure 5a,b). At both excitation wavelengths, the intensity of the main MB fluorescence peak at 696 nm decreases in the following order: V20→V80→S20→S80. This sequence differs from the normalized absorbance shown in Figure 4: V20→S20→V80→S80. This indicates that after illumination new species appear in the solutions, which contribute differently to absorbance and luminescence. When excited by light with a wavelength of 320 nm, the maximum emission bands are observed in the range of 450–460 nm (Figure 5a). Fluorescence at ~450 nm for *leuco*-MB and ~460 nm for *leuco*-Thionine (Th) has been reported upon excitation at a wavelength of 320 nm. At both excitation wavelengths (320 and 365 nm), the luminescence peak observed at 696 nm for an unilluminated MB solution gradually shifts toward a shorter wavelength up to approximately 680 nm. This is due to the demethylation of the MB molecule [22]. According to the results of FTIR spectroscopy measurements, the decrease in the intensity of the 690 nm peak may be associated with several decomposition stages occurring in different parts of the MB molecular [46]. Sequential demethylation results in the formation of structurally related by-products such as Asure B, Azure A, Azure C, and thionine [47,48,49], which causes charge redistribution and a shift in electron density to the nitrogen atom in the central aromatic ring. This electron transition causes luminescence at ~550 nm upon 365 nm excitation [33], which can be seen in Figure 5b. The peaks at 565 and 570 nm are also associated with changes in the aromatic rings of the MB molecule [22,33]. Thus, the emission maxima observed at 560–580 nm upon excitation at a wavelength of 365 nm (Figure 5b) indicate a complex stepwise degradation of the MB molecule.

The SOS peaks marked with asterisks for the respective excitation wavelengths (Figure 5a,b) are shown at a larger scale in Figure 5c,d. The intensity of the SOS peaks at 640 nm after excitation at a wavelength of 320 (Figure 5c) decreases in the same order as the intensity of the luminescence peaks (Figure 5a). Note that the intensity of the SOS peaks is higher than the SOS peak of the unilluminated MB solution. This indicates an increase in the number of scattering centers after irradiating the MB solution since the intensity of the SOS peak correlates with the concentration of the scattering centers, but the intensity of the luminescence of these centers is significantly lower than the luminescence of the MB molecule. At an excitation wavelength of 365 nm, an intense SOS peak at 730 nm is observed only in a solution illuminated with sunlight at 80 °C (S80) for 120 min. The strong dependence of light scattering on the excitation wavelength is confirmed by the fact that the SOS spectra have a pronounced maximum at a certain wavelength [39]. 

### 3.3. BNO_x_ Photocatalyst

**Figure 6:**
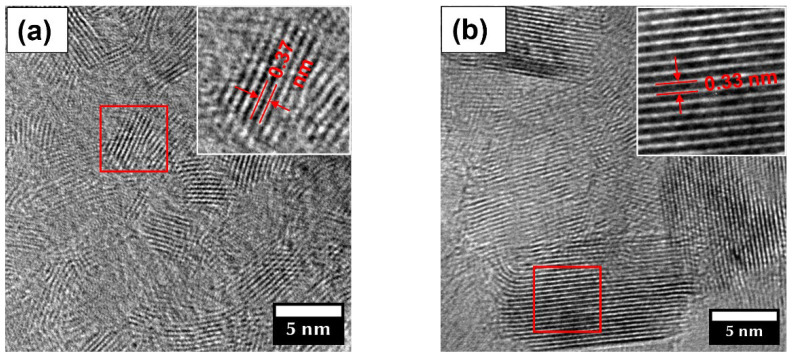
TEM images of the BNO_1_ (**a**) and BNO_2_ (**b**) samples. Insets show enlarged areas marked with red rectangles (red lines and arrows mark atomic planes).

BNO_x_ NPs with differing oxygen content were studied as photocatalysts. NPs designated BNO_1_ were synthesized using the low-temperature ammonolysis of boric acid at a sintering temperature of 650 °C, as described elsewhere [23]. Sample BNO_2_ was prepared by annealing a portion of the BNO_1_ powder in ammonia at 1100 °C for 1 h. Figure 6 represents TEM images of the BNO_1_ (a) and BNO_2_ (b) samples.

The TEM analysis shows that the BNO_1_ and BNO_2_ samples are composed of nanocrystals with an average size of approximately 5 and 10 nm, respectively. Insets show enlarged areas marked with red rectangles. An interlayer spacing was determined to be 0.37 and 0.33 nm for the BNO_1_ and BNO_2_ samples, respectively.

**Figure 7:**
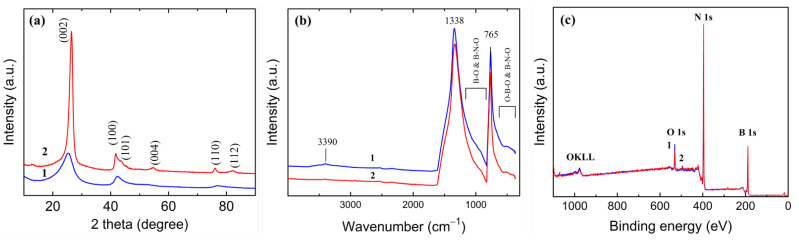
XRD patterns (Cu-Kα radiation) (**a**), FTIR (**b**), and XPS (**c**) spectra of the BNO_1_ (1) and BNO_2_ (2) nanopowders.

Figure 7 represents XRD patterns: the FTIR and XPS spectra of these samples are denoted as 1 and 2 for BNO_1_ and BNO_2_, respectively.

The XRD analysis shows that sample BNO_2_ is nanocrystalline *h*-BN (Figure 7a, curve 2). The interlayer spacing along the *c*-axis is 0.33 nm, and the average crystal size, determined by the Debye–Scherrer equation, is approximately 10 nm. The XRD pattern of sample BNO_1_ shows broadened, low-intensity peaks typical of the turbostratic *h*-BN structure [23,50]. This sample has an interlayer distance along the *c*-axis of 0.35 nm and an average crystal size of 6 nm. The XRD data are in good agreement with the results of the TEM analysis. The FTIR spectrum of sample BNO_2_ (Figure 7b) demonstrates an intense peak at 1338 cm^−1^ due to in-plane B-N stretching vibrations and a narrow peak at 765 cm^−1^ due to out-of-plane B-N-B bending vibrations. The absence of other peaks indicates an almost pure BN phase. The FTIR spectrum of sample BNO_1_ (Figure 7b) demonstrates a small peak at 3390 cm^−1^, attributed to asymmetric O-H stretching vibrations or an overtone of B-O trigonal vibrations, and shoulders in the range of 1200–840 cm^−1^ and 670–400 cm^−1^ assigned to B-O stretching vibrations and B-O-B and B-N-O bending vibrations, respectively [23]. The XPS analysis (Table 1) shows that samples BNO_1_ and BNO_2_ consist of boron, nitrogen, and oxygen in the following amounts: 6.5 (1) and 4.2 at. % (2). The nitrogen content of sample BNO_1_ is lower than the boron content, which indicates that oxygen mostly substitutes nitrogen rather than boron. Indeed, in oxidized BN, oxygen atoms substitute nitrogen atoms and form B-O bonds [51]. A more detailed description of the initial structure and its transformations during heat treatment can be found elsewhere [23].

**Figure 8:**
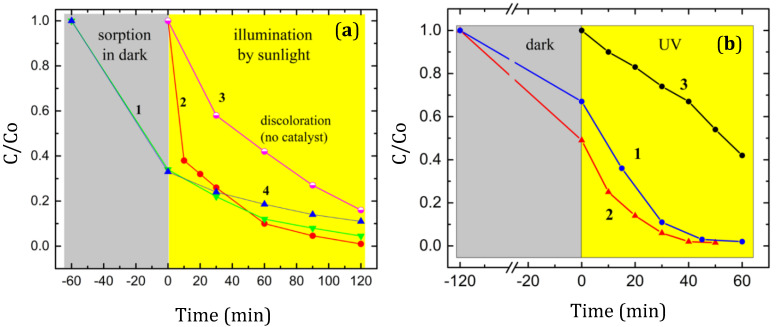
Time-dependences of the normalized absorbance of MB solutions (0.5 mg MB in 50 mL H_2_O) containing 10 mg of BNO_1_ under sunlight illumination at 80 °C after 1 h of sorption in the dark (curve 1) and without sorption in the dark (curve 2). For comparison, the discoloration of the MB solution without a catalyst is shown (curve 3). Curve 4 shows the photocatalytic degradation of the solution under visible light illumination (**a**). Time-dependences of the normalized absorbance of MB solutions (0.5 mg MB in 50 mL H_2_O) containing 5 mg of BNO_1_ (curve 1) and 5 mg of BNO_2_ (curve 2) under UV illumination at 80 °C after 2 h of sorption in the dark (**b**). Curve 3 shows the discoloration of the MB solution under UV irradiation without a catalyst.

To study the effect of MB sorption on MB degradation, two measurements were carried out: with sorption in the dark for an hour followed by sunlight illumination (curve 1 in Figure 8a) and with sunlight without sorption in the dark (curve 2 in Figure 8a). For comparison, the discoloration curve of the MB solution without a catalyst is also shown (curve 3 in Figure 8a).

The results obtained show that the discoloration rate without the sorption stage in the dark (curve 2 in Figure 8a) of the BNO_1_-containing MB solution is higher than the discoloration rate after sorption in the dark. This implies that the sorption of MB molecules on the BNO_1_ surface deactivated some of the active centers involved in the photodegradation process. Within one hour of illumination, the discoloration of the MB solution reached 90%. A comparison with curve 3 in Figure 8a (without a catalyst) clearly shows that the discoloration occurs not only due to the MB photocatalytic degradation, but also due to the discoloration of the MB solution itself, and ignoring this fact introduces a significant error to the assessment of the photocatalytic activity of the catalyst. The specific catalyst mass activity was calculated taking into account the discoloration of the MB solution under sunlight according to the following equation:

(C_3_(t) − C_2_(t)) × m_MB_/m_cat_/Δt (1)

where C_2_ and C_3_ are the normalized absorbance values of the MB solution with a photocatalyst (curve 2) and without a photocatalyst (curve 3) at time t, Δt is the solution irradiation time, and m_MB_ and m_cat_ are the masses of the MB and catalyst in solution. The specific photocatalytic mass activity of the BNO_1_ nanopowder during the photodegradation of an MB solution (10 mg·L^−1^) under sunlight illumination for an hour was calculated to be 15 mg·g^−1^·h^−1^ (50 μmol·g^−1^·h^−1^). Note that, without taking into account the MB solution discoloration, the catalyst activity would be three times higher.

**Figure 9:**
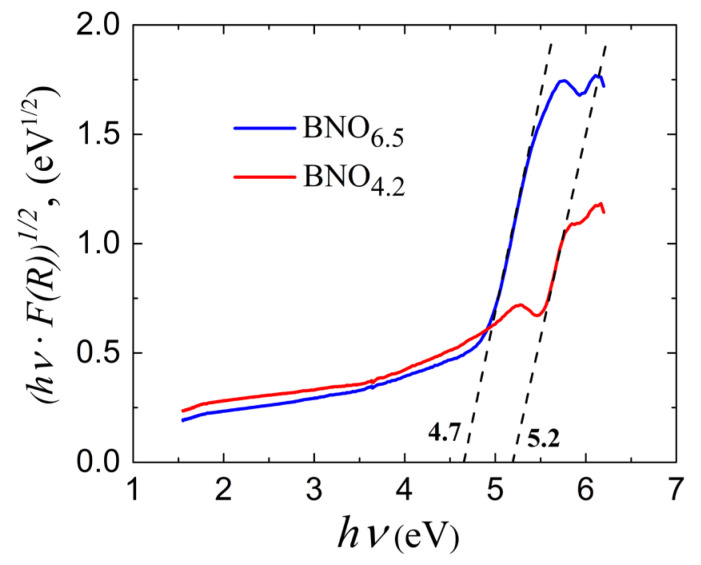
Tauc plots of the BNO_1_ and BNO_2_ nanopowders containing 6.5 and 4.2 at.% of oxygen.

Figure 8b shows the time-dependent discoloration of MB solutions containing BNO_1_ and BNO_2_ NPs under UV illumination after MB sorption in the dark (curves 1 and 2). An estimate of the band gap values in samples BNO_1_ and BNO_2_ based on the diffuse reflectance spectra (Figure 9) yielded 4.7 and 5.2 eV, respectively. The energy of the generated UV photons using a low-pressure mercury lamp generating UV light at a wavelength of 254 nm was 4.88 eV. This value exceeds the band gap of sample BNO_1_ and is large enough to excite electrons from the valence band to the conduction band. However, in the case of sample BNO_2_, with a band gap of 5.2 eV, the energy of the UV photons is insufficient for the direct generation of photoelectrons. The DFT simulation (see below) shows that, when an MB molecule is adsorbed on the BNO_x_ surface, the nitrogen atom of the central MB ring forms a strong chemical bond with the boron atom closest to the oxygen defect. This causes a redistribution of the electron density near the oxygen defect and leads to the formation of an interband state. Thus, it can be assumed that MB sorption on the surface of BNO_x_ NPs leads to the formation of additional levels near the conduction band, which provide photoexcitation with lower energy photons.

Curve 3 in Figure 8b shows the discoloration of the MB solution (without a catalyst) under UV irradiation. As with exposure to sunlight, there was a strong discoloration (about 60%) within an hour. Approximately the same discoloration rate was observed under sunlight for an hour, but in these experiments, the catalyst was taken twice as much (10 and 5 mg for sunlight and UV experiments, respectively). Therefore, the discoloration rate under UV illumination is twice as high as under sunlight. Discoloration is a complex process and includes the following mechanisms: thermally induced MB photodegradation, MB absorption on BNO_x_ NPs, self-sensitizing MB photooxidation, and photocatalytic MB degradation. Careful consideration of all these processes makes it possible to determine the photocatalytic contribution to the discoloration process, and in this case, MB can be used as a test material.

Taking all MB discoloration mechanisms into account, and using Equation (1), the specific photocatalytic mass activity of the BNO_1_ and BNO_2_ nanopowders during half an hour of illumination was calculated to be ~140 mg·g^−1^·h^−1^ (440 μmol·g^−1^·h^−1^). Photocatalytic activity in the MB degradation of various catalytic systems is shown in Table 1.

Pure BN is an indirect semiconductor with a bandgap of about 6 eV, which is much higher than the photon energy of visible light. Doping with oxygen reduces the band gap, and with a high oxygen content, it can be only 2.1 eV [52]. To estimate the band gaps of the BNO_1_ and BNO_2_ nanopowders, diffuse reflectance spectra (DRS) were collected. Figure 9 shows a Tauc plot,

((*F*(*R*_∞_) × *hν*)^1/γ^ = *B*(*hν* − *E*_g_),

converted from DRS using the Kubelka–Munk function (*F*(*R*_∞_)),

$$F\left(R_{\infty }\right)=\frac{{\left(1-R_{\infty }\right)}^{2}}{2R_{\infty }},$$

where *R*_∞_ = *R*_sample_/*R*_standart_ is the reflectance of an infinitely thick specimen, *h* is Planck’s constant, *ν* is the photon frequency, *E*_g_ is the band gap energy, and *B* is a constant. For the indirect semiconductors, γ = 2 [53].

From the Tauc plot, the band gap energies of samples BNO_1_ and BNO_2_ were determined to be 5.2 and 4.7 eV, respectively. Since these values exceed the energy of visible light, it was assumed that the observed photocatalytic activity of BNO_1_ is associated with the presence of a UV component in the sunlight spectrum. To evaluate this effect, the photocatalytic degradation of the MB solution in the presence of the BNO_1_ nanocatalyst was measured under visible light illumination (curve 4 in Figure 8a). It can be seen that the degradation rate slightly decreased. Note that a noticeable photodegradation of an MB aqueous solution was observed under irradiation with a laser beam with a wavelength of 670 nm [54]. It has been suggested that the photobleaching of an MB aqueous solution is a photodynamic process [54,55,56,57], and MB is a powerful photosensitizer that generates reactive oxygen species (ROS), including singlet oxygen ^1^O_2_ and superoxide anion **^-^**O**˙**_2_ [58,59]. The ROS generated during MB photosensitization can attack the material itself and lead to photochemical reactions on its surface (the so-called self-sensitized photooxidation). This explains the discoloration of the MB solution under visible or solar light illumination.

**Figure 10:**
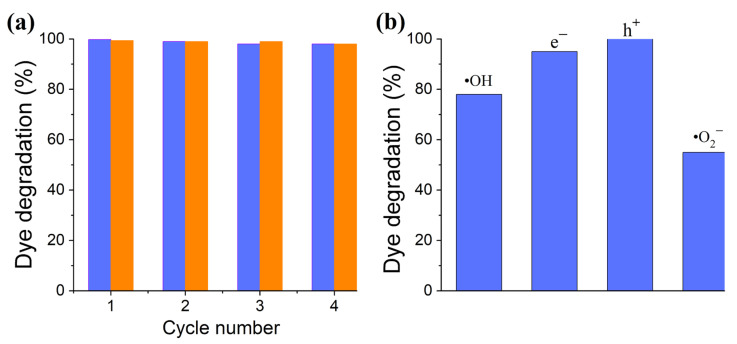
Photocatalytic activity and stability of BNO_1_ and BNO_2_ nanoparticles during the degradation of MB under UV irradiation (45 min) in four successive cycles (blue and orange colors correspond to 6.5 and 4.2 at.% of oxygen) (**a**); results of scavenger testing of the BNO_1_ sample (**b**).

To assess the stability and reusability of the BNO_x_ nanocatalysts, four successive cycles of degradation of MB solutions under UV illumination were carried out. The obtained results are shown in Figure 10a. After four cycles, the degradation ability of the catalysts remained at the 98% level, which indicates their high stability.

In the process of the photodegradation of organic dyes in the presence of a wide-gap photocatalyst, the following main reaction stages are usually considered [60,61,62]:

(2) $$\mathrm{Catalyst}+h\nu \;\to \;e_{CB}^{-}+h_{VB}^{+}$$

(3) $$(O_{2}{)}_{\mathrm{ads}}+e_{CB}^{-}\;\to {\;}^{-}O{˙}_{2}$$

(4) $$H_{2}O+h_{VB}^{+}\;\to \;H^{+}+\cdot \mathrm{OH}$$

**^-^**O**˙**_2_ + H^+^ → **·**OOH (5)

**·**OOH + **·**OOH → H_2_O_2_ + O_2_ (6)

H_2_O_2_ → 2**·**OH (7)

organic dye + **·**OH → CO_2_ + H_2_O (8)

The band gap of BN is large enough for the photolytic formation of superoxide radicals (**·**O_2_^−^) from adsorbed oxygen. According to reaction (2), BN absorbs UV light and generates electrons in the conduction band and holes in the valence band as charge carriers. The electrons interact with adsorbed oxygen to form superoxide radicals (3). The holes interact with the H_2_O molecule adsorbed on the BN surface to form a hydrogen ion and hydroxyl radicals (4). The superoxide radical (2) interacts with a hydrogen ion (4) to form hydroperoxy radicals (5) and, hence, generates hydrogen peroxide and molecular oxygen (6). Hydrogen peroxide is then decomposed into hydroxyl radicals by UV irradiation (7). Hydroxyl radicals are strong oxidants and decompose organic dyes (8).

To determine the primary reaction in the MB photodecomposition process in the presence of the BNO_1_ catalyst, scavenger tests were performed (Figure 10b). The addition of a hole scavenger did not affect the photodegradation process, while the electron scavenger only slightly reduced the degradation efficiency. In contrast, scavengers of **·**OH and, especially, **^-^**O**˙**_2_ species significantly reduced the degradation kinetics. Accordingly, we assume that these radicals make the main contribution to the dye degradation process. It was mentioned above that the superoxide anion **^-^**O_2_ is also generated by MB. Thus, in an MB-BNO_x_ system, the superoxide anion is generated via both processes: MB photosensitization and BNO_x_-catalyzed photolysis. This explains its great contribution to MB photodegradation.

Experiments on the photocatalytic UV degradation of MB solutions (Figure 8b) show that the sorption capacity of BNO_x_ NPs depends on the oxygen content, and as it increases from 4.2 to 6.5 at.% (curve 1), the sorption capacity decreases. This is exactly the opposite of what one would expect since the specific surface area of BNO_1_ is almost 1.4 times higher than that of BNO_2_ (122.56 and 89.89 m^2^·g^−1^). The XPS analysis (Table 1) showed that the nitrogen content in the BNO_2_ sample increased relative to boron, while the oxygen content decreased. Obviously, high-temperature annealing in ammonia led to the substitution of nitrogen for part of the oxygen, and the substitution mainly affected the oxygen atoms located on the surface. This means that the lower sorption capacity of the BNO_1_ sample is associated with higher oxygen content on the surface. In this regard, it should be noted that surface oxygen defects do not change activity, since the photocatalytic activity of both materials is almost the same (Figure 8b). During photocatalysis, a photocatalyst is also exposed to the active particles formed, which usually leads to its oxidation and degradation. The high stability of the BNO_2_ photocatalyst is expected until it is oxidized to an oxygen content comparable to sample BNO_1_.

As can be seen from the comparison catalysts in Table 2, BNO_x_ NPs are an efficient photocatalyst for MB degradation under UV irradiation.

### 3.4. Computational Analysis of MB Sorption on BNO_x_

As noted above, oxygen defects on the BNO_x_ surface prevent the sorption of MB molecules. At first glance, this is surprising since MB exists in the solution as a cation, and one would expect increased sorption due to negatively charged oxygen substituents. To elucidate the sorption process of MB molecules on BNO_x_, we calculated the sorption energy depending on the orientation of the MB molecule using DFT. A layer of oxidized BN (6.5 at.% of O) was used as a model system. During the simulation, various possibilities for the location of the MB molecule on the BN surface were considered. The sorption process of the MB molecule on the oxidized BN differs from the process on pure *h*-BN. In the case of a defect-free BN surface, a flat MB molecule stands on an edge at an angle of about 45 degrees to the plane. This orientation makes it possible to create a denser packing and, as a result, increases the sorption capacity of BN with respect to BNO_x_.

**Figure 11:**
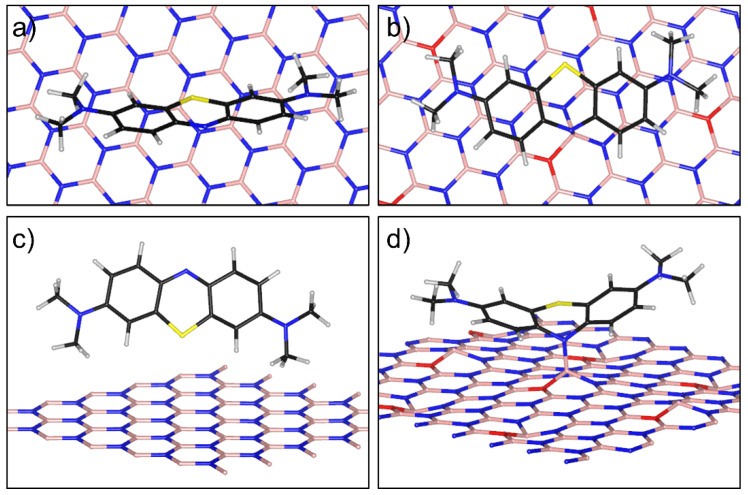
Methylene blue molecule adsorbed on the surface of pure (**a**,**c**) and oxidized BN (**b**,**d**), top (**a**,**b**) and side (**c**,**d**) view, respectively. Oxygen defects are marked in red and nitrogen in blue.

In the case of BNO_x_, the MB molecule is oriented parallel to the surface so that its aromatic system is above the BN rings (Figure 11a). This orientation is most likely due to the mutual coordination of conjugated π-systems over each other. In addition, the result of our simulation showed that the nitrogen atom of the central MB ring is bound to the boron atom nearest to the oxygen defect and forms a chemical bond with a bonding energy of 2.7 eV and a bond length of 1.55 Å (Figure 11b), which is 0.1 Å less than the B-N bond in BN. Thus, the MB molecule strongly binds to the surface of the oxidized BN, as evidenced by both the distance between the atoms and the binding energy of the molecule to the surface. A strong bond is possible due to the redistribution of the charge on the MB molecule and the redistribution of the electron density near the oxygen defect in the BN. Despite the strong chemical binding, the location of the MB molecule is such that it occupies a large surface area of the BNO_x_, which, accordingly, reduces its sorption capacity.

## 4. Conclusions

The optical absorbance of methylene blue (MB) aqueous solutions in a concentration range of 0.5 to 10 mg·L^−1^ and photolytic effects leading to discoloration of MB solutions with and without boron oxynitride (BNO_x_) nanoparticles (NPs) were studied under various types of illumination (visible light, sunlight, and UV light). It was shown for the first time that in an MB concentration range of 3.33 to 4.00 mg·L^−1^, there is a violation of the linear dependence of optical absorption on the MB concentration, which is due to the formation of dimeric and trimeric molecular associates. This must be taken into account in order to correctly assess the MB concentration. The fractions of the monomer, dimer, and trimer in the MB solution with a concentration of 10 mg·L^−1^, estimated from the absorption peak areas, are approximately 91.1%, 7.1%, and 1.8%, respectively.

The MB solutions discolorized when they were illuminated in a wide spectral range, from visible light to the UV-B range (254 nm). This process is thermally dependent, and the discoloration rate in visible light and sunlight nearly doubles as the temperature rises from 20 to 80 °C. MB discoloration may be due to its self-sensitized photooxidation, in which MB, when illuminated, generates reactive oxygen species that oxidize MB molecules. Although thermally induced MB discoloration has been demonstrated only at 20 and 80 °C, it is clear that it occurs at any temperature in this range, but with less efficiency. This effect can be easily exploited in practice, either by using an excess of industrial heat or with focused sunlight.

A DFT analysis of MB sorption capacity on BNO_x_ NPs shows that surface oxygen defects prevent the sorption of MB molecules. This is due to the planar orientation of the MB molecule above the BNO_x_ surface. The calculations also show that the MB molecule is chemically bound to the BNO_x_ surface by the boron atom nearest to the oxygen defect. A strong electrostatic interaction changes the electronic configuration of the MB molecule and increases its self-sensitizing activity. This explains the enhanced photodegradation of MB in visible light in the presence of BNO_x_ nanoparticles.

The discoloration process of MB involves the following mechanisms: thermally induced MB photodegradation, MB absorption on BNO_x_ nanoparticles, self-sensitizing MB photooxidation, and photocatalytic MB degradation. Accounting for all these processes makes it possible to reveal the contribution of the photocatalyst to the discoloration process, and in this case, MB can be used as a test material.

Taking into account all these mechanisms of MB discoloration, the photocatalytic activity of BNO_x_ NPs containing 4.2 and 6.5 at.% of oxygen was studied under UV irradiation of MB aqueous solutions. The specific mass activity of both types of NPs is approximately 140 mg·g^−1^·h^−1^ (440 μmol·g^−1^·h^−1^). The high photocatalytic activity of BNO_x_ NPs in a wide range of oxygen substitutions, combined with their high stability, makes them promising metal-free photocatalysts for water treatment.

## Figures and Tables

**Table 1:** Elemental composition of BNO_x_ samples synthesized at 650 (BNO_1_) and 1100 °C (BNO_2_). The rest is carbon from adsorbed carbonaceous species.

Sample	Content, at. %		
	B	N	O
BNO_1_	48.0	44.8	6.5
BNO_2_	47.7	46.6	4.2

**Table 2:** Photodegradation of MB over various photocatalysts under UV irradiation.

Photocatalyst	Light Source	%MB Degraded@Time	Reference
CuO/Bi_2_O_3_ Nanocomposite	UV-C irradiation	88.32%@120 min	[60]
5% PTh/ZnO	250 W high-pressure mercury lamp	95%@180 min	[61]
ZnO-NR/ACF Nanocomposites	UV irradiation	99%@120 min	[62]
γ-Fe_3_/Fe_3_O_4_/SiO_2_ (Ar modified)	UV irradiation	87.5%@120 min	[63]
70% CeO_2_/g-C_3_N_4_ Z-scheme Heterojunction	UV irradiation	90.1%@180 min	[64]
BNO_x_ nanoparticles	50 W low-pressure mercury lamp	100%@45 min	This work

## Data Availability

There are no data to report.
